# Are ECG changes in heart-healthy individuals of various ages related to cardiac disease 20 years later?

**DOI:** 10.48101/ujms.v126.6064

**Published:** 2021-05-21

**Authors:** Sofia Erelund, Kjell Karp, Urban Wiklund, Rolf Hörnsten, Sandra Arvidsson

**Affiliations:** a Department of Surgery and Perioperative Sciences, Umeå University, Umeå, Sweden; b Department of Radiation Sciences, Umeå University, Umeå, Sweden

**Keywords:** Heart function test, electrocardiogram, normal values, clinical physiology, arrhythmia

## Abstract

**Background:**

This research study aimed at assessing the electrocardiographic (ECG) changes caused by ageing in a cohort of healthy subjects with normal echocardiographic examinations.

**Methods:**

A total of 219 healthy individuals (119 males and 100 females) were evaluated for possible arrhythmias with a standard 12-lead resting ECG and 24-h Holter ECG. As the recordings were performed between 1998 and 2000, a 20-year follow-up study was carried out by assessing the local medical records to investigate whether the subjects had experienced any cardiovascular health complications or disease since the baseline assessment.

**Results:**

Eighty-three subjects (45 males and 38 females) presented with pathological ECG findings at baseline. The most common finding on analysis of Holter ECG recordings was premature atrial contractions, and the most severe pathological finding was episodes of ventricular tachycardia (eight subjects). Regarding the analysis of the standard 12-lead ECG, the most common finding was left ventricular hypertrophy, and the most severe pathological findings were ST-T changes and prolongation of the QT interval. Despite other cardiac examinations performed on these patients showing normal results, in combination with a strict inclusion criterion, this study showed that 28% of all subjects had pathological resting 12-lead ECGs at rest and 35% had pathological heart rhythms when assessed by 24-h Holter ECG. At follow-up, 21% of females and 43% of males had presented with ECG abnormalities, and 30% of females and 36% of males had cardiovascular disease. There was hypertension in 45% of females and in 58% of males. However, no association was found between the follow-up findings and ECG changes seen at baseline.

**Conclusion:**

Although most ECG changes found at baseline could be considered as a normal variation, they may progress to more severe heart complications as the subject ages. The results of this study also validate ECG findings of previous studies and underline that diagnostic criteria should be based on gender and age.

## Introduction

Today, people are living longer than ever before, and therefore, it has become even more important to distinguish between normal ageing-related cardiovascular changes and pathological cardiovascular conditions. In order to detect abnormal findings in a patient population, healthy controls of both genders and of a wide range of ages are required. Controls are often randomly selected from the population and included if they pass an initial clinical investigation and fit the selection criteria. Such a control group was included in a general population study in Umeå, Sweden, where the aim was to identify normal cardiovascular changes due to ageing in otherwise healthy subjects, with a particular focus on determining reference values for use in echocardiographic (echo) examinations of patients (1). The following study focuses on the age-related changes in the electrocardiographic (ECG) recordings that were carried out on the same controls, but not have been published before: 12-lead resting ECG and 24-h Holter ECG monitoring. We also performed a 20-year follow-up study to assess for development of cardiovascular health complications based on a review of medical records.

Minor ECG changes do not necessarily prevent a subject from being included in an echo reference material (2). Moreover, previous studies in which healthy adults have been screened with Holter ECGs have revealed that arrhythmias are common in the elderly population (3–7). Although most of the changes have been considered as benign, other studies have shown that a minor change in ECG also increases the risk of having more severe heart complications in later life (8–11). To evaluate the presence of pathological ECG changes in this cohort, we applied the standard criteria used at our clinic: the Minnesota Code for resting ECGs, and pathological Holter ECGs were assessed according to the criteria defined by Bjerregaard (12).

We also compared the resting ECG parameters in this general population with another reference material from the Netherlands (2), which included 13,354 healthy subjects, aged 16–90 years, with no evidence of cardiovascular disease. This is of interest as both age-related and gender-dependent changes in ECG parameters have been published previously.

This study aimed to describe age-related changes in ECG carried out in the cohort of healthy subjects from Northern Sweden and to compare our findings with previously published normal values. Data from 196 standard 12-lead ECGs and 112 Holter ECGs from healthy subjects of age 20–90 years were analysed. In addition, a 20-year follow-up study was conducted in order to assess whether ECG changes observed at baseline were related to development of cardiovascular disease.

## Material and methods

Data for this study originate from a local general population heart study performed in Umeå, Sweden during the period 1998–2000 (1). From the Tax Agency’s birth records, 1,000 subjects (1:1, male: female) were randomly selected from 15 pre-determined years of birth (1905,1910,1915,1920 up to 1975). These subjects received a letter containing information about the study’s content and purpose, as well as a request to participate. The inclusion criteria were assessed by a phone interview and included the absence of any known cardiovascular or systemic disease and no use of medications that could possibly affect the heart function. The upper limit of normal blood pressure was defined as 160/90 mmHg based on the wide range of ages. A specially designed questionnaire was sent to all eligible subjects before the investigations began. Subjects with diabetes, hypertension, hyperlipidaemia, stroke, previous transient ischemic attack, rheumatic fever and/or intermittent claudication were excluded, resulting in a total of 300 subjects.

The previously published study was solely focusing on echo data. In this study, ECGs were also recorded in 219 subjects (119 males and 100 females). Of those, 196 performed standard 12-lead ECG, 112 performed Holter ECG and 89 subjects performed both examinations. Analysis of these investigations was performed by specialised medical technicians and doctors. This study was approved by the Regional Ethics Committee in Umeå (2017-537-32M, addition to 98-129). All subjects were coded, and the analysis was blinded. All participants gave their written informed consent for participation. The procedures followed were in accordance with the Declaration of Helsinki of 1964, as revised, and with the good clinical practice guidelines.

### Standard 12-lead ECG

A standard 12-lead ECG was performed with the subject in the supine position and at rest using six precordial and six extremity leads. The electrode placement was as follows: black – right leg, green – left leg, red – right arm, yellow – left arm. Precordial: V1 in the fourth intercostal space at the right sternum edge; V2 in the fourth intercostal space at the left sternum edge; V3 diagonally between V2 and V4; V4 in the fifth intercostal space in the medio clavicular line; V5 at the height of V4, in the anterior axillary line; and V6 in height with V4 and V5 in the middle axillary line. A paper speed of 50 mm/s was used, as well as a gain setting of 10 mm range for a test signal of 1 mV. The leads were presented in the Cabrera format (13).

Standard 12-lead ECGs were interpreted according to the Minnesota code Manual (14). The normal heart rate (HR) for adults is defined to be in the range of 50–100 beats per minute (bpm). Normal rhythm was defined as a sinus rhythm with positive P waves in lead I, II, avF and V2–V6. The P wave should be positive with a normal amplitude of <2.5 mm and a duration of <120 ms. The normal PQ interval is defined to be in the range of 120–200 ms and <220 ms for subjects older than 60 years. The normal Q-wave duration was defined as <0.03 s. QRS interval was considered normal if <110 ms. The ST segment was measured at the J point in leads V1, V5 and V6, and was considered pathological if the elevation (horizontal or downward sloping) was ≥0.1 mV. The normal QT interval was defined to be in the range of 390–460 ms for women and 390–450 ms for men. Corrected QT (QTc, Bazzet’s correction) was calculated and was considered as abnormal if QTc was >460 ms for women and >450 ms for men. T waves were considered to be normal if they were positive in leads I, II, and V3–V6. Left ventricular hypertrophy (LVH) was considered as present if the R + S amplitude was >35 mm. The cardiac axis was considered to lie between −30° and +90° and was measured in the lead with the largest R-wave amplitude.

The measured ECG parameters were compared with reference values published in a study by Rijnbeck et al. (2), where the median, 2 and 98% percentiles for 10-year age intervals were defined.

### Long-term ECG

Holter ECG recordings were performed using the Braemer DL 700 digital ECG recorder (Braemer Inc., Burnsville, MN, USA). Three channels (V1, V3 and V5) were recorded by placing five colour-coded electrodes on the patient’s chest. The analysis was performed using the Aspect Holter system (GE Healthcare Sverige AB, Danderyd, Sweden) software.

The Holter ECGs were evaluated with respect to rhythm, presence and frequency of premature atrial contractions (PACs) and premature ventricular contractions (PVCs), paired or in series, episodes of bradycardia or tachycardia, atria-ventricular block I–III (AV-Block), sino-atrial block or sinus arrest, right or left bundle branch block (LBBB, RBBB), and atrial fibrillation (AF) or flutter.

The evaluation of the Holter ECGs was performed according to Bjerregaard (4). The following events were defined as pathological: lowest heart rate <40 bpm (based on 1 min); AV block I-III; sinoatrial blocks / sinus arrest > 2 sec; >10 PACs/24 h in subjects 20–40 years; >100 PAC/24 h in subjects 40–60 years; >1,000 PAC/24 h in subjects >60 years; more than two different morphologies of PAC; paroxysmal supraventricular tachycardia in subjects <50 years; two or more episodes of SVT or SVT with more than 10 consecutive beats in subjects > 50 years; >100 PVCs/24 h in subjects <50 years; >200 PVC/24 h in subjects > 50 years; more than two morphologies of PVC; single pairs of PVC in subjects <50 years; PVC in bigeminy; R on T PVC; ventricular tachycardia (VT) (> 3 PVC consecutive and heart rate > 100 beats/min); and AF or flutter.

### 20-year follow-up

The follow-up study was performed using the local medical record system, Evry NCS care portal, version 5.11.8.1 (Evry Sweden AB, Eskilstuna, Sweden). Each subject’s medical record was reviewed from the date of inclusion to follow-up (autumn 2019). Data on diagnostic codes and inpatients were taken into account, as well as data from visits to outpatient clinics and health centres within the region. During the follow-up period, 13 subjects had moved to another region of Sweden, and therefore, were excluded from the follow-up. The follow-up findings were classified as follows: (1) cardiovascular disease and (2) ECG abnormalities. Cardiovascular disease included myocardial infarction, heart failure, angina pectoris, stroke, trans-ischemic attack, intermittent claudication and valvular heart diseases. The subgroup ‘ECG abnormalities’ included conduction disorders, supraventricular and/ or ventricular arrhythmias and if they had received pacemaker therapy. In addition, the presence of hypertension, diabetes and vital status (alive or deceased) were noted.

### Statistical analysis

Data processing and statistical analyses were performed in Microsoft Office Excel 2016 (Microsoft corporation Redmond WA USA), MATLAB (MathWorks Inc., Natick, MA, USA) and SPSS version 27 (IBM, Armonk, NY, USA). The subjects were divided into three different groups based on the following age: <50, 50–65 and >65 years. Chi-square tests were used for comparisons between the frequency of arrhythmic events for different age ranges in the pathological group. Mean and standard deviation were calculated for all ECG parameters, and two-way analysis of variance (ANOVA) using a linear regression model was used for comparison of females, males and age groups. There was no interaction between age group and sex. Chi-square tests were used for comparison of baseline and follow-up findings. The null hypothesis was rejected for *P* < 0.05.

## Results

### Standard 12-lead ECG

A standard 12-lead ECG was recorded in 196 subjects, of which 54 subjects (28%) presented with pathological findings (Table 1). In both genders, the majority of pathological ECG findings were reported in subjects >60 years, where 38% of females and 37% of males had presented with pathological findings. Table 2 shows a summary of rhythm and conduction disorders, as well as abnormal morphology in subjects with a pathological standard 12-lead ECG. The most common conduction disorder reported was AV-block I. Subjects >65 years presented with more conduction disorders than the expected proportion in this age group. More frequent rhythm disturbances than the expected proportion were found in the age group <50 years. Ten out of the 12 individuals who presented with rhythm disorders had bradycardia episodes, one with a nodal rhythm pattern and one with AF. Abnormal morphology was most frequently found in the age group > 65 years. The most common form of abnormal ECG morphology was LVH.

**Table 1:** Pathological events in male and female subjects with standard a 12-lead electrocardiogram (ECG).

ECG findings	Females/males		
	<50 years (N 23/30)	50–65 years (N 22/36)	> 65 years (N 42/43)
Pathological ECG	4/10	3/5	16/16
Left ventricular hypertrophy	0/4	1/1	4/6
P-wave pathology	0/0	2/1	0/2
Q-wave pathology	0/0	0/0	3/2
Deviating QRS morphology	0/1	0/0	2/2
S-wave pathology	1/0	0/1	0/0
ST-T changes	0/3	0/0	5/1
T-wave pathology	0/0	0/0	6/2
Long QT-time	1/0	1/0	1/1
Short PQ-time	1/0	0/0	0/1
Atria-ventricular-block I	0/0	1/1	3/4
Left or right anterior fascicular block	0/0	1/2	1/2
Right bundle branch block or left bundle branch block (RBBB/LBBB)	0/0	0/1	2/2
Left axis deviation	0/0	0/0	2/3
Bradycardia	2/4	1/0	1/2
Nodal rhythm	0/1	0/0	0/0
Atrial fibrillation	0/0	0/0	0/1

**Table 2:** Summary of rhythm disturbances, conduction disturbances and abnormal morphology in subjects with a pathological standard 12-lead electrocardiogram (ECG).

ECG findings	Females/males		
	<50 years (N 23/30)	50–65 years (N 22/36)	>65 years (N 42/43)
Rhythm disturbances	2/5*	1/0	1/3
Conduction disturbances	1/0	2/3	5/8*
Abnormal morphology	2/8	2/3	12/11*

* *P* < 0.05 Chi-square test.

Tables 3 and 4 present the ECG characteristics in subjects with normal standard 12-lead ECGs. A significant age-dependency was found in HR, PQ-interval, QTc and P-wave duration. Compared to females, males presented with an increased PQ-interval duration, QRS duration, P-wave duration and R+S amplitude.

**Table 3:** Mean (SD) of variables from the standard 12-lead resting electrocardiogram (ECG) in three different age groups for heart-healthy females.

Resting ECG without remark	< 50 years (*n* = 19)	50–65 years (*n* = 19)	> 65 years (*n* = 26)
Heart rate (beats/min)	64 (11)	67 (10)	68 (11)*
PQ-time (ms)	153 (26)^†^	174 (36)*^†^	170 (22)*^†^
QRS duration (ms)	87 (10)^†^	87 (11)^†^	78 (13)^†^
QT-time (ms)	383 (26)	388 (48)	389 (28)
QTc (ms)	392 (29)	408 (41)*	412 (26)*
P-wave duration (ms)	84 (20)^†^	93 (15)*^†^	92 (15)*^†^
P-wave amplitude (mV)	0.19 (0.07)	0.16 (0.05)	0.16 (0.04)
R+S (mV)	2.2 (0.5)^†^	2.3 (0.6)^†^	2.4 (0.6)^†^

* *P* < 0.05 compared with <50 years.

† *P* < 0.05 compared with males.

**Table 4:** Mean (SD) of variables from a standard 12-lead resting electrocardiogram (ECG) in three different age groups for heart-healthy males.

Resting ECG without remark	< 50 years (n = 20)	50–65 years (n = 31)	> 65 years (n = 27)
Heartrate (beats/min)	60 (12)	64 (7)	68 (13)*
PQ-time (ms)	169 (20)^†^	178 (21)*^†^	176 (25)*^†^
QRS-duration (ms)	92 (8)^†^	86 (16)^†^	91 (10)^†^
QT-time (ms)	380 (30)	378 (30)	391 (39)
QTc (ms)	378 (30)	391 (32)*	412 (32)*
P-wave duration (ms)	91 (16)^†^	100 (13)*^†^	102 (23)*^†^
P-wave amplitude (mV)	0.15 (0.04)	0.16 (0.05)	0.16 (0.05)
R+S (mV)	2.5 (0.6)^†^	2.6 (0.5)^†^	2.6 (0.5)^†^

* *P* < 0.05 compared with <50 years.

† *P* < 0.05 compared with females.

**Figure 1:**
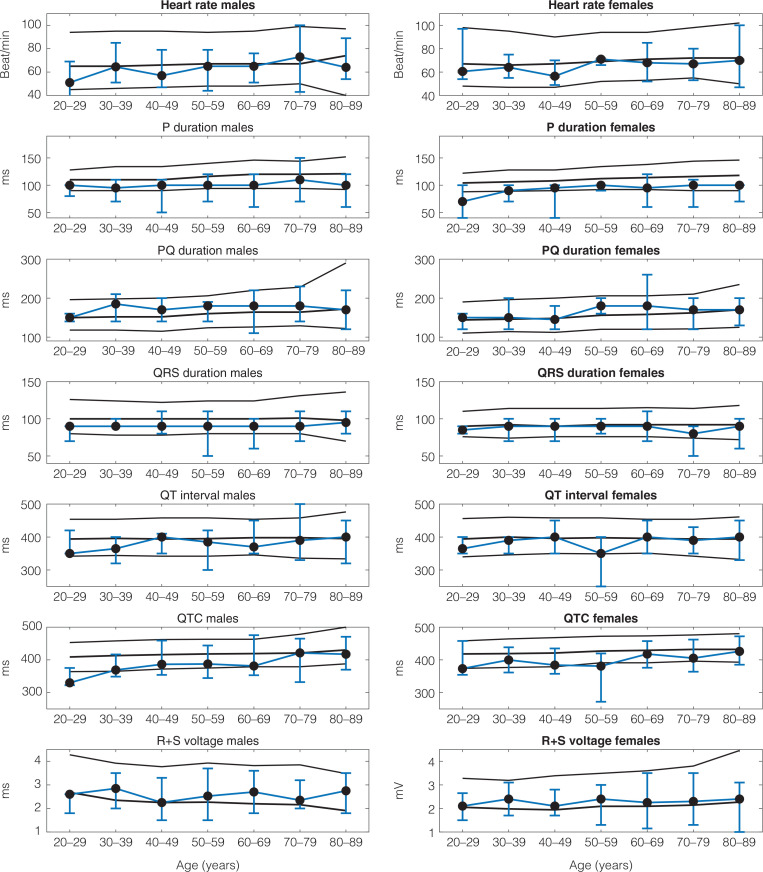
Electrocardiographic (ECG) parameters from the 12-lead ECG in 10-year intervals. Solid lines show reference values (2), presented as median as well as 2 and 98% percentiles. Error bars show data from this study, presented as median, minimum and maximum.

A comparison of the measured ECG parameters with the previously published reference values is shown in Figure 1, where lines indicate median, 2 and 98% percentile for the reference values. Error bars show our data, with minimum, maximum and median values, on fewer individuals in each 10-year interval than in the reference material. Most ECG characteristics compared well with the reference values; however, the P-wave duration tended to be shorter in this study.

### Long-term ECG

The 24-h Holter ECG recordings were performed in 112 subjects (56 females, 56 males), of which 39 subjects (35%, 22 females, 17 males) presented with the pathological findings (Table 5). Subjects > 50 years were over-represented in regard to findings, such as > 2 episodes of SVT (7 females and 8 males) and > 200 PVC/24 h (6 females and 7 males). Eight subjects (4 females and 4 males) > 50 years had episodes of VT, and 4 females and 2 males > 50 years, and 1 female <50 years had episodes of PVC bigeminy. Four subjects, 2 females > 50 and 2 males <50 had AV-block II type I.

**Table 5:** Classification of abnormal findings according to Bjerregard’s classification (4). tested on 112 healthy individuals with 24-h Holter electrocardiogram (ECG).

Abnormal findings	Number of individuals (female/male)
10 premature atrial contraction (PAC)/24 h: <40 years	1 (1/0)
100 PAC/24 h: 40–59 years	2 (0/2)
1000 PAC/24 h: >60 years	6 (3/3)
Paroxysmal supraventricular tachycardia (SVT): <50 years	1 (1/0)
> 2 episodes of SVT: >50 years	15 (7/8)
> 100 premature ventricular contractions (PVC)/24 h: <50 years	2 (2/0)
>200 PVC/24 h: >50 years	13 (6/7)
single pairs of PVC: <50 years	4 (3/1)
PVC in bigeminy	7 (5/2)
R on T PVC	1 (1/0)
Ventricular tachycardia	8 (4/4)
Atria-ventricular-block II type 1	4 (4/0)

PVCs were observed in 95 subjects (85%, 48 females, 47 males), where 47 subjects presented with more than 10 PVC/24 h, of which 42 subjects were >50 years. Eighteen subjects (10 females, 8 males) had more than 100 PVC/24 h.

### Overall ECG findings

Table 6 shows a summary of pathological findings in two different types of ECG recordings according to age and gender. Thirty-eight percentage of both males and females had presented with pathological findings in either one or both of the recordings. The highest proportion (50%) of pathological events in the Holter ECG was observed in both genders >65 years. The lowest proportion (6%) of pathological events was observed on Holter ECG recordings in males <50 years, where 33% had pathological standard ECGs. This was mainly due to episodes of bradycardia as presented above.

**Table 6:** Summary of pathological electrocardiographic (ECG) recordings according to sex and age.

ECG findings	Pathological/number of recordings			
	<50 years	50–65 years	>65 years	All
**Females**				
Pathological ECG	9/29 (31%)	6/28 (21%)	23/43 (53%)	38/100 (38%)
Pathological standard ECG	4/23 (17%)	3/22 (14%)	16/42 (38%)	23/87 (26%)
Pathological long-term ECG	6/18 (33%)	5/16 (31%)	11/22 (50%)	22/56 (39%)
**Males**				
Pathological ECG	11/38 (29%)	13/36 (36%)	21/45 (47%)	45/119 (38%)
Pathological standard ECG	10/30 (33%)	5/36 (14%)	16/43 (37%)	31/109 (28%)
Pathological long-term ECG	1/18 (6%)	8/22 (36%)	8/16 (50%)	17/56 (30%)

### 20-year follow-up

At follow-up, the medical records of 206 (104 females) subjects were examined. Of those, 57% of females and 65% of males were still alive. ECG abnormalities were observed in 21% of females and 43% of males. Cardiovascular diseases were reported in 30% of females and 36% of males. Hypertension was observed in 45% of females and 58% of males. In all participants regardless of the baseline ECG status, there was development of more severe ECG abnormalities, as well as cardiovascular diseases (Table 7).

**Table 7:** Summary of findings at follow-up according to sex, age and electrocardiographic (ECG) findings at baseline examination.

Findings at follow-up	Number of individuals (normal or pathological ECG at baseline)			
	<50 years	50–65 years	>65 years	All
**Females**				
Number of follow-ups	27 (18/9)	25 (19/6)	52 (26/26)	104 (63/41)
Alive	27 (18/9)	20 (14/6)	12 (7/5)	59 (39/20)
Cardiovascular disease	0	7 (4/3)	24 (12/12)	31 (16/15)
ECG abnormalities	1 (0/1)	5 (2/3)	16 (5/11)	22 (7/15)**
Hypertension	4 (2/2)	15 (9/6)	28 (16/12)	47 (27/20)
Diabetes	2 (1/1)	1 (1/0)	3 (2/1)	6 (4/2)
**Males**				
Number of follow-ups	29 (23/6)	38 (26/12)	35 (17/18)	102 (66/36)
Alive	28 (22/6)	30 (19/11)	8 (3/5)	66 (44/22)
Cardiovascular disease	1 (1/0)	12 (9/3)	24 (11/13)	37 (21/16)
ECG abnormalities	2 (2/0)	13 (9/4)	29 (14/15)	44 (25/19)
Hypertension	12 (10/2)	18 (12/6)	29 (14/15)	59 (36/23)
Diabetes	2 (1/1)	1 (1/0)	7 (4/3)	19 (6/4)

** *P* < 0.01, Chi-square test.

## Discussion

In this research study, we have assessed ECG changes related to ageing in a cohort of healthy subjects who presented with normal results in other cardiac examinations. We found significant age- and gender-related changes in many ECG parameters; however, pathological changes in ECG were observed in all age groups. In the youngest age group, bradycardia and LVH were frequently observed, whereas in the oldest age-group, conduction disturbances and morphological changes were more commonly found in the resting ECG. Rhythm disturbances were most frequently observed in the Holter ECG studies in subjects >50 years. In the resting ECG, rhythm disturbances were most frequently observed in the subjects <50 years, and the most common type was bradycardia.

At the 20-year follow-up study, 30% of all females and 36% of all males had developed cardiovascular disease; however, there was no statistically significant difference between those who presented with pathological ECGs at baseline and those with normal baseline ECGs. ECG abnormalities were found in 21% of all females, and although they were significantly more common in those with pathological baseline ECGs than in those with normal baseline ECGs, no association between baseline and follow-up findings was noted within any of the three age groups. In 43% of males, ECG abnormalities were observed at follow-up; however, there was no relation with baseline ECG findings.

As in previous studies (2, 15, 16), our subjects with ECGs without pathological findings presented with differences between genders and changes that were consistent with ageing. Both age- and gender-related differences were noted in the P-wave duration and in PQ interval. QRS duration and R+S only presented with differences related to gender. For HR and QTc, differences were only observed between age groups. ECG changes related to ageing have been discussed for a long time and may be related to a change in heart placement that occurs with ageing, which, in turn, can be due to changes in weight, subcutaneous fat distribution, diaphragm position, thorax and lung parenchyma. This may also explain why there is a gender difference in the ECGs (2).

We also compared the results of this study with the large study from the Netherlands by Rinjbeck et al. (2), in which several variables matched well with the Dutch reference material. The highest mean HR (68 bpm) was observed in both males and females > 65 years, which is similar to the findings of Rinjbeck et al. (2). In this study, we noted a tendency to shorter P-duration compared with the reference material. The mean QT duration obtained in this study was marginally lower in all age groups than that found by Rinjbeck et al. In this study, four subjects presented with a prolonged QT duration, and QTc was extended in the older age groups. This, however, can be due to medical treatments, such as antibiotics, antidepressants and antihistamines (17).

Eight subjects (4%) presented with T-wave changes, which were found among both males and females >65 years. There are T-wave changes in several disease states, for example, in myocardial ischemia, and should not occur in a healthy individual. Therefore, T-wave abnormalities should be given a special attention and be correlated with clinical information (18).

ECG findings indicating LVH were reported in 16 subjects (8%) and across all age groups. LVH occurs when the heart is continuously exposed to pressure overload, for example, in hypertension, aortic stenosis or due to hard physical exercise. One of the exclusion criteria in this cohort was hypertension, and since the subjects did not receive any medical treatment for heart disease, blood pressure should not have been the main cause for the signs of LVH in this study.

The presence of PVC in 24-h Holter ECGs can indicate an underlying cardiac pathology. In this study, 85% of all subjects presented PVC, with a range of 1–12,800 PVC/24 h. However of these, only 13% fell within the range of what is classified as pathological. Martin et al. studied apparently healthy subjects aged > 75 years and found that the death rate at 3 years was twice as high in subjects having > 10 PVC/h in a 24-h Holter ECG (10). Frishman et al. stated that 93% of the subjects had PVC, but with a low incidence of non-sustained VT (11). In this study, eight subjects (7%) had VT, which can occasionally occur in healthy subjects. However, this should not be considered as normal, and subjects presenting with VT should always be further examined.

No significant relationships were found between the findings at follow-up and the presence of a pathological ECG at baseline; however, we cannot exclude that minor changes in ECG can be a predictor of more severe cardiac events, as has been reported in previous studies (8–11). A total of 26 subjects (11 females and 15 males) experienced a myocardial infarction in the 20 years between baseline and follow-up, of these four were aged between 50 and 65 years and the others were >65 years. In this study, 13 males and 9 females developed heart failure, and all except one male were in the age group > 65 years. As expected, few in the oldest age groups were alive. The cause of death has not been determined in this study, but many previous studies (19–21) have reported an increased mortality in those who had an abnormal ECG. Therefore, it cannot be ruled out that a part of this population has died of cardiovascular diseases. A few deaths were observed in the age groups under 65 years, where 86% of the males and 90% of the females were alive.

At follow-up, hypertension was observed in 51% of all subjects. The incidence of hypertension increased with ageing, and by retirement age, 54% of the females and 83% of the males had a high blood pressure. These findings can be compared with the MONICA study from Northern Sweden, where the incidence of hypertension was 42% in subjects aged 50–59 years, 62% in those 60–69 years and 73% in subjects 70–79 years (22).

The main limitation of this study is that not all subjects underwent both resting 12-lead and Holter ECG studies at baseline. Additionally, the upper blood pressure limit stipulated at the time of subject recruitment,160/90 mmHg, is today considered hypertensive, and therefore, some of the pathological results may be due to hypertension at baseline. The follow-up was performed by only assessment of the local medical records. Access to national medical databases would have been advantageous in order to add more information and follow more subjects who had left the region.

In conclusion, to correctly assess cardiac disorders for different genders and ages with a diagnostic method such as ECG, there should be gender- and age-specific reference values.

Twenty-eight percentage of the subjects who underwent a 12-lead standard ECG presented with pathological results, and 35% of all Holter ECGs presented with pathological findings. Although our 20-year follow-up study revealed that most of the changes found at baseline can be considered as benign, they may develop to more severe heart complications as the subject ages.
